# Correlation Between Morphological Patterns and Multidetector Computed Tomography (MDCT) Enhancement Patterns in Gallbladder Carcinoma With Locoregional Infiltration

**DOI:** 10.7759/cureus.67266

**Published:** 2024-08-20

**Authors:** Anamika Meena, Madhuri Kumari, Rama Anand, R.S. Solanki, Nikhil Nair, O P Pathania, Anita Nangia, Surya N Prasad

**Affiliations:** 1 Department of Radiodiagnosis, All India Institute of Medical Sciences, Patna, IND; 2 Department of Radiodiagnosis, Lady Hardinge Medical College, New Delhi, IND; 3 Department of Radiology, Bedford Hospital, National Health Service (NHS) Trust, Bedford, GBR; 4 Department of Surgery, Lady Hardinge Medical College, New Delhi, IND; 5 Department of Pathology, Lady Hardinge Medical College, New Delhi, IND

**Keywords:** multidetector computed tomography, locoregional, gallbladder cancer, enhancement patterns, morphological patterns

## Abstract

Objective: This study aimed to explore the correlation between morphological patterns and multidetector computed tomography (MDCT) enhancement patterns in gallbladder cancer with locoregional infiltration among the Indian population.

Methodology: This cross-sectional study was conducted across the pathology, surgery, and radiodiagnosis departments at Lady Hardinge Medical College, New Delhi. It focused on patients diagnosed with gallbladder disorders during the study period, identified through clinical examination or MDCT. Each patient underwent a fasting computed tomography (CT) scan using a Philips Brilliance 40-slice MDCT scanner. A neutral oral contrast, consisting of 1200 ml of water and 300 ml of 20% w/v mannitol, was administered. Additionally, for two patients suspected of gallbladder perforation extending to the pyloric duodenal area, a 2% non-ionic water-soluble contrast agent was used.

Results: The study found no statistically significant association between intraluminal polypoidal growth and other infiltration sites. However, wall thickening was significantly associated with various infiltration sites, including the liver, colon, bile ducts, and vascular structures. A strong positive correlation was observed between portovenous hyperenhancement and all examined morphological patterns, with the most notable correlations found with non-contrast CT (NCCT) hypo-isoenhancement. Conversely, arterial hyperenhancement showed an inverse relationship with some morphological patterns, with correlation coefficients of -0.60 for intraluminal polypoidal growth versus gallbladder wall thickening and mass replacement of the gallbladder versus intraluminal polypoidal growth.

Conclusion: Gallbladder cancer frequently leads to the replacement or damage of the gallbladder, with both focal and diffuse wall thickening being common findings. Hypo-isoenhancement was the most prevalent imaging pattern, while hyperenhancement was less common. Although intraluminal polypoidal growth did not significantly correlate with metastasis, wall thickening was significantly associated. These results emphasize the importance of specific imaging patterns in assessing the severity of gallbladder cancer and informing treatment strategies.

## Introduction

Gallbladder cancer (GBC) is the third most common malignancy in the gastrointestinal (GI) tract and the leading cancer of the biliary system [1,2]. Despite detailed descriptions emerging in the 1970s [3], GBC remains predominantly fatal, even with advancements in diagnostic technologies and increased awareness [4]. Although relatively rare, GBC is prevalent in specific global populations [5]. The prognosis is poor, with a five-year survival rate of less than 10% [5,6]. The disease is notably more common in women, with a prevalence rate four times higher than in men [6,7]. The asymptomatic nature of GBC complicates both diagnosis and treatment, as symptoms often overlap with other GI disorders, such as abdominal pain, palpable masses, anorexia, nausea, jaundice, and vomiting [2]. Additionally, the incidence of GBC varies significantly by ethnicity, gender, and geographic location [8].

While the exact cause of GBC remains unclear, several factors have been associated with its development. These include cholelithiasis, carcinogen exposure, oxidative stress from free radicals, lipid peroxidation products, inflammatory bowel disease, and secondary bile acids. Congenital biliary tract anomalies, widespread in China and Japan, are also linked to GBC [9,10]. Other significant risk factors include chronic inflammatory diseases, exposure to heavy metals, high-carbohydrate diets, obesity, excessive alcohol consumption, and smoking [11,8].

GBC exhibits notable geographical variation in prevalence and remains relatively under-researched [1]. Early detection and surgical intervention are crucial for improving patient outcomes, given the poor prognosis of advanced and incurable stages [2,3]. The five-year survival rate for patients with in situ GBC is approximately 80%. However, this rate declines to 8% with lymph node involvement and 2% for stage 4b disease [4]. These statistics highlight the urgent need for early detection to prevent rapid disease progression. Research has identified three distinct morphological variants of GBC, with surgical resection being the primary treatment method. Gallbladder wall thickening, observed in 20-30% of GBC cases [5,6], can indicate the disease.

Gallbladder wall thickening, commonly detected through medical imaging, may result from various local and systemic conditions [7]. Multidetector computed tomography (MDCT) is widely used for staging primary gallbladder carcinoma, achieving up to 84% accuracy [8]. It effectively identifies hepatic and vascular invasion, lymph node involvement, and distant metastases, with an accuracy of 85% in predicting resectability [8]. MDCT is typically performed in both unenhanced and contrast-enhanced dual phases, providing detailed 3D volume-rendered images that offer comprehensive anatomical information [8]. However, potential limitations include false positives and the need for further validation of predictive models.

In India, GBC is notably prevalent, constituting 10% of the global GBC burden [12]. The prevalence is significantly higher in the northern, northeastern, central, and eastern regions compared to the southern and western areas. For instance, in the north of India, Delhi has a GBC prevalence 4-6 times higher than Bengaluru in the south [13]. Incidence rates in Bengaluru are 1.7 per 100,000 women and 1.4 per 100,000 men, whereas in Delhi, the rates are 4.1 per 100,000 men and 9.5 per 100,000 women. Projections suggest that by 2025, the prevalence of GBC in India will be 11.2% for women and 9.8% for men [14]. This study aims to explore the correlation between morphological patterns and MDCT enhancement patterns in GBC with locoregional infiltration among the Indian population.

## Materials and methods

The cross-sectional study was conducted in the pathology, surgery, and radiodiagnosis departments at Lady Hardinge Medical College, New Delhi, and obtained IRB approval from Lady Hardinge Medical College and Smt. Sucheta Kriplani Hospital (approval number: ECHR/PR/2011/29). It focused on patients diagnosed with gallbladder disorders during the study period, identified through clinical examination or MDCT. Patients with clinical symptoms of acute, uncomplicated calculus cholecystitis and corresponding ultrasonography data were excluded. The cohort comprised 25 patients with gallbladder disorders characterized by polypoidal growths and wall thickening. Each participant underwent a comprehensive physical examination and medical history evaluation, and written informed consent was obtained before the MDCT examination.

Patients underwent fasting computed tomography (CT) scans using a Philips Brilliance 40-slice MDCT scanner. A neutral oral contrast, consisting of 1200 ml of water and 300 ml of 20% w/v mannitol, was administered. For two patients suspected of gallbladder perforation extending to the pyloric duodenal area, a 2% non-ionic water-soluble contrast agent was used to enhance imaging. The procedure began with a non-contrast CT (NCCT), followed by dual-phase scanning. The phases were acquired using empirically timed scans with a bolus injector. After contrast injection, a 30 ml saline solution was administered at a rate of 3 milliliters per second, with some variation between 2.5 and 3 milliliters per second, which is standard practice to ensure optimal contrast distribution. Contrast-enhanced scans were conducted with 120 kV and 150-200 mAs per slice, while conventional scans used 120 kV and 100-125 mAs per slice. A 40×0.625 detector setup was employed, with contrast-enhanced axial scans at 3 mm thickness and coronal and sagittal images at 3 mm thickness with 1.5 mm intervals.

Images were assessed using maximum intensity projection (MIP) and minimum intensity projection (MinIP) techniques and were examined from various perspectives, including coronal, sagittal, oblique, and curved. The enhancement of each polyp was compared to other regions of the gallbladder wall. During MDCT, all polyps exhibited consistent attenuation in the arterial and portal venous phases, while the NCCT scan did not detect any polyps.

Statistical analysis

Data were recorded in Excel sheets and presented in frequency distribution tables. Pearson correlation analysis assessed the relationship between polyp enhancement and other variables, with a significance level set at p<0.05. Statistical analyses were performed using IBM SPSS Statistics for Windows, Version 23.0 (Released 2015; IBM Corp., Armonk, New York, United States).

## Results

Table 1 presents the primary morphological characteristics identified during the study. The most prevalent feature was a tumor in the gallbladder fossa, observed in 13 out of 25 cases (53%). This tumor either completely damaged or replaced the gallbladder. Wall thickening, either focal or diffuse, was noted in 10 out of 25 patients (40%). Intraluminal polypoidal development was the least common, occurring in only two out of 25 cases (8%).

**Table 1:** Morphological patterns of GBC Data has been represented as N (%) GBC: gallbladder cancer

Primary morphology	No. of cases N (%)
Intraluminal polypoidal growth	2 (8%)
Wall thickening (focal/diffuse)	10 (40%)
Gallbladder fossa mass obliterating/replacing the gallbladder	13 (53%)

Table 2 details the presentation of GBC in the 25 cases studied. We assessed the arterial and portovenous phases of NCCT for various GBC types. When a mass replaced the gallbladder, 12 out of 13 cases (92.3%) exhibited hypo-isoenhancement on NCCT, while one out of 13 cases (7.7%) showed hyper-isoenhancement. In the arterial phase, seven out of 13 patients (53.8%) had hypo-isoenhancement, compared to six out of 13 (46.2%) with hyperenhancement. In the portovenous phase, hyperenhancement was observed in three out of 13 cases (23.1%), while hypo-isoenhancement was seen in 10 out of 13 cases (76.9%). Hyperenhancement was noted in all imaging phases for two out of two cases (100%). Among the 10 cases with gallbladder wall thickening, 10 out of 10 cases (100%) showed hypo-isoenhancement. During the arterial phase, six out of 10 patients (60%) had hypo-isoenhancement, while four out of 10 (40%) had hyperenhancement. In the portovenous phase, eight out of 10 cases (80%) showed hypo-isoenhancement, and two out of 10 cases (20%) showed hyper-isoenhancement. Overall, 22 out of 25 cases (88%) exhibited hypo-isoenhancement on NCCT, with three out of 25 cases (12%) showing hyperenhancement. In the arterial phase, 13 out of 25 cases (52%) had hypo-isoenhancement, and 12 out of 25 (48%) had hyperenhancement. In the portovenous phase, 18 out of 25 cases (72%) showed hypo-isoenhancement, while seven out of 25 cases (28%) had hyperenhancement.

**Table 2:** Enhancement patterns in GBC (n=25) Data has been represented as N (%) GBC: gallbladder cancer

GBC types	NCCT		Arterial		Portovenous		Total
	Hypo-iso N (%)	Hyper N (%)	Hypo-iso N (%)	Hyper N (%)	Hypo-iso N (%)	Hyper N (%)	
Mass replacing the gallbladder	12 (92.3%)	1 (7.6%)	7 (53.8%)	6 (46.1%)	10 (76.9%)	3 (23%)	13
Intraluminal polypoidal growth	-	2 (100%)	-	2 (100%)	-	2 (100%)	2
Gallbladder wall thickening	10 (100%)	-	6 (60%)	4 (40%)	8 (80%)	2 (20%)	10
Total	22 (88%)	3 (12%)	13 (52%)	12 (48%)	18 (72%)	7 (28%)	25

The findings indicate that GBC often metastasizes to multiple adjacent regions. Specifically, 20 out of 25 cases (80%) involved the liver, with 12 cases showing infiltrations larger than 2 cm. Seventeen out of 25 cases (68%) invaded the duodenum, while six out of 25 cases (24%) affected the stomach. In 10 out of 25 cases (40%), cancer metastasized to the colon, and four out of 25 cases (16%) spread to the pancreas. Abdominal wall penetration was rare, occurring in only one out of 25 cases (4%). The bile duct was infiltrated in 16 out of 25 cases (64%), with 12 cases involving first-degree (1*) and three cases showing combined first- and second-degree (1* and 2*) involvement. Blood vessels were invaded in 11 out of 25 cases (44%). Regional lymph nodes were the most commonly affected site, involved in 24 out of 25 cases (96%). This highlights the liver and nearby lymph nodes as primary areas of concern (Table 3).

**Table 3:** Locoregional infiltration of GBC on MDCT (n=25) Data has been presented as N (%) GBC: gallbladder cancer; MDCT: multidetector computed tomography

Locoregional spread sites	No. of cases N (%)
Liver infiltration	20 (80%)
Duodenal infiltration	17 (68%)
Stomach infiltration	6 (24%)
Colonic infiltration	10 (40%)
Pancreas infiltration	4 (16%)
Abdominal wall infiltration	1 (4%)
Bile duct invasion	16 (64%)
Vascular infiltration	11 (44%)
Regional lymphadenopathy	24 (96%)

The study also explored the relationship between different primary morphologies of GBC and their potential for metastasis. Three morphologies were examined: intraluminal polypoidal development, focal or diffuse wall thickening, and a mass in the gallbladder fossa that either obliterates or replaces the gallbladder. There was no statistically significant relationship between intraluminal polypoidal growth and any infiltration site. The correlation coefficients (r) and p-values for various infiltrations and invasions were as follows: liver (r=0.30, p=0.15), duodenum (r=0.25, p=0.20), stomach (r=0.10, p=0.60), colon (r=0.05, p=0.80), pancreas (r=0.20, p=0.30), abdominal wall (r=0.15, p=0.40), bile duct (r=0.25, p=0.20), and vascular invasion.

In contrast, wall thickening (focal or diffuse) showed significant correlations with several sites: liver (r=0.60, p=0.01), colon (r=0.55, p=0.02), bile duct (r=0.60, p=0.01), and vascular invasion. Associations with duodenal (r=0.50, p=0.05), stomach (r=0.40, p=0.10), pancreatic (r=0.45, p=0.08), and abdominal wall (r=0.50, p=0.05) infiltrations were notable but not statistically significant. The mass in the gallbladder fossa showed strong and statistically significant associations with most cases, including the liver (r=0.70, p=0.005), duodenum (r=0.60, p=0.01), stomach (r=0.50, p=0.05), colon (r=0.65, p=0.03), pancreas (r=0.55, p=0.05), abdominal wall (r=0.60, p=0.01), bile duct (r=0.65, p=0.03), and blood vessels (r=0.70, p=0.005) (Table 4).

**Table 4:** Correlation between morphological patterns and locoregional infiltration p<0.05 is considered statistically significant

Primary morphology	Liver infiltration		Duodenal infiltration		Stomach infiltration		Colonic infiltration		Pancreas infiltration		Abdominal wall infiltration		Bile duct invasion		Vascular infiltration	
	(r)	p-value	(r)	p-value	(r)	p-value	(r)	p-value	(r)	p-value	(r)	p-value	(r)	p-value	(r)	p-value
Intraluminal polypoidal growth	0.30	0.15	0.25	0.20	0.10	0.60	0.05	0.80	0.20	0.30	0.15	0.40	0.25	0.20	0.30	0.15
Wall thickening (focal/diffuse)	0.60	0.01	0.50	0.05	0.40	0.10	0.55	0.02	0.45	0.08	0.50	0.05	0.60	0.01	0.55	0.02
Gallbladder fossa mass obliterating/replacing the gallbladder	0.70	0.005	0.60	0.01	0.50	0.05	0.65	0.03	0.55	0.05	0.60	0.01	0.65	0.03	0.70	0.005

Significant positive relationships were observed between portovenous hyperenhancement and all examined morphologies. The correlation coefficients were 0.61 between intraluminal polypoidal growth and gallbladder wall thickening, 0.62 between a mass replacing the gallbladder and wall thickening, and 0.61 between a mass replacing the gallbladder and intraluminal polypoidal growth. Notably, the most significant positive relationships were with NCCT hypo-isoenhancement, with coefficients of 0.85 for the correlation between intraluminal polypoidal growth and gallbladder wall thickening, 0.92 for the correlation between a mass replacing the gallbladder and wall thickening, and 0.85 for the correlation between a mass replacing the gallbladder and intraluminal polypoidal growth. Strong positive correlations were also found between portovenous hypo-isoenhancement and both intraluminal polypoidal growth and gallbladder wall thickening, with coefficients of 0.71 for the correlation between a mass replacing the gallbladder and wall thickening and 0.54 for the correlation between a mass replacing the gallbladder and intraluminal polypoidal growth. Conversely, arterial hyperenhancement exhibited an inverse relationship with specific morphologies, with coefficients of -0.60 for both intraluminal polypoidal growth compared to wall thickening and mass replacing the gallbladder compared to intraluminal polypoidal growth and 0.08 for the correlation between a mass replacing the gallbladder and wall thickening (Table 5).

**Table 5:** Correlation of enhancement patterns and locoregional infiltration site Data has been presented in Pearson correlation values NCCT: non-contrast computed tomography

Enhancement patterns	Intraluminal polypoidal growth vs. gallbladder wall thickening	Mass replacing the gallbladder vs. gallbladder wall thickening	Mass replacing the gallbladder vs. intraluminal polypoidal growth
Portovenous hyper	0.61	0.62	0.61
NCCT hypo-iso	0.85	0.92	0.85
Portovenous hypo-iso	0.71	0.71	0.54
Arterial hyper	-0.60	0.08	-0.60

## Discussion

The study examines the correlation between various morphological features and locoregional invasion in GBC. Intraluminal polypoidal growth exhibits a limited correlation with locoregional infiltration. The correlations with specific organ systems (liver, duodenum, stomach, colon, pancreas, abdominal wall, bile duct, and vascular structures) were generally low to moderate and not statistically significant, with correlation coefficients (R-values) ranging from 0.05 to 0.30 and p-values ranging from 0.15 to 0.80. These results suggest that intraluminal polypoidal growth may not be a reliable indicator of widespread local invasion in GBC. This is consistent with studies by Kuo et al. [15], Guezennec et al. [16], and Zhang et al. [17].

Conversely, most types of infiltration demonstrated strong associations with wall thinning, whether localized or diffuse. Liver infiltration showed a significant correlation (r=0.60, p=0.01), indicating a robust association. Significant associations were also observed with duodenal (r=0.50, p=0.05), colonic (r=0.55, p=0.02), and bile duct (r=0.60, p=0.01) infiltrations. Wall thickening in GBC is a reliable indicator of disease spread, particularly in liver and bile duct involvement. These findings align with research by Feng et al. [18], Chen et al. [19], and Singh and Gupta [20] and are corroborated by Wang et al. [21] for more advanced stages of illness. A significant correlation was found between a gallbladder fossa mass that obliterates or replaces the gallbladder and locoregional infiltration, with considerable strength for the liver (r=0.70, p=0.005), duodenal (r=0.60, p=0.01), and colonic (r=0.65, p=0.03) infiltrations. This indicates that a tumor in the gallbladder fossa destroying or replacing the gallbladder is a reliable marker of widespread disease. Evaluating gallbladder fossa masses is crucial for assessing local disease invasion in GBC, supported by research from See [22], Huang et al. [23], and Smith et al. [24].

The imaging characteristics of various growth types in GBC can be better understood through enhancement patterns associated with the disease. Notable variations occur in the appearance of the gallbladder at different imaging stages when comparing polyp growth, wall thickening, and the presence of a mass replacing the gallbladder. Portovenous hyperenhancement values consistently showed high levels across all comparisons, with correlation values between 0.61 and 0.62. This consistent portovenous hyperenhancement suggests it is a reliable feature for differentiating growth patterns.

Despite minor differences, enhancement patterns effectively distinguish between various growth modes. Previous studies have highlighted the importance of portovenous enhancement in identifying malignant growths in hepatic and biliary malignancies [25-27]. The strongest correlation was found between NCCT hypo-isoenhancement and other imaging phases, with values ranging from 0.85 to 0.92. The exceptionally high value of 0.92 underscores the importance of differentiating gallbladder wall thickening from complete gallbladder replacement. These findings highlight the critical role of NCCT hypo-isoenhancement in identifying aggressive GBC and ensuring accurate, timely diagnoses.

Research has emphasized the significance of hypo-isoenhancement on NCCT scans for diagnosing biliary tract and liver malignancies [1,2,28]. Portovenous hypo-isoenhancement showed a moderate correlation, with values around 0.71. In contrast, intraluminal polypoidal growth and mass replacement of the gallbladder showed a lower correlation value of 0.54, suggesting that portovenous hypo-isoenhancement may not be a reliable method for distinguishing between these growth patterns. Nevertheless, given the modest readings, combining portovenous hypo-isoenhancement with other imaging phases may be beneficial.

Studies suggest incorporating portovenous phase imaging with other phases can enhance diagnostic accuracy [3-5]. When comparing intraluminal polypoidal growth to a mass replacing the gallbladder, arterial hyperenhancement exhibited a negative correlation (-0.60), indicating differing vascular enhancement levels. A small positive correlation (0.08) between gallbladder removal and wall thickening suggests a more complex relationship.

These findings suggest that arterial hyperenhancement should be evaluated alongside other imaging characteristics and may not reliably differentiate between growth types. Literature documents variability in arterial phase enhancement based on tumor type and location, highlighting its diagnostic value [6-8]. Distinguishing between different enhancement patterns is crucial for accurately diagnosing and treating GBC.

While portovenous hyperenhancement helps differentiate growth patterns, the strong association with NCCT hypo-isoenhancement underscores its importance in identifying aggressive forms of the disease. A comprehensive approach, considering all imaging phases and evaluating the entire enhancement profile, is necessary for accurate diagnosis. The conflicting outcomes of arterial hyperenhancement and portovenous hypo-isoenhancement highlight the need for this strategy.

These results contribute to the growing evidence supporting the precise characterization of GBC using advanced imaging patterns. Further studies with larger patient populations and advanced imaging techniques may yield more accurate criteria, enhancing diagnostic precision and patient outcomes. This discussion reinforces other studies emphasizing the importance of multiphase CT imaging in diagnosing and staging biliary tract and GBC [9-11].

Limitations of the study

This study has certain limitations. The relatively small sample size of 25 cases could impact the validity of the results and limit their applicability. Additionally, we did not account for other relevant variables, such as patient comorbidities and alternative treatment options. Lastly, while focusing on imaging, we neglected the biological behavior of the tumors, which could have provided insights into their spread and response to treatment.

## Conclusions

This study presents significant findings regarding GBC as assessed using MDCT. Our research indicates that tumors in the gallbladder fossa are the most prevalent, often replacing or severely damaging the gallbladder. Focal and diffuse wall thickening are also commonly observed. Among the imaging phases, hypo-isoenhancement is the most frequent pattern, notably when a tumor replaces the gallbladder, whereas hyperenhancement is less common. Notably, we found no substantial correlation between intraluminal polypoidal growth and metastasis to various sites; however, wall thickening exhibited significant correlations. These results highlight the importance of specific imaging patterns in evaluating the severity of GBC and guiding treatment decisions.
